# Detection of CSF1 gene derangement in ‘sclerosing mucoepidermoid carcinoma with eosinophilia’ of the parotid gland masquerading as Langerhans cell histiocytosis

**DOI:** 10.1002/ccr3.7488

**Published:** 2023-06-07

**Authors:** Florence Man Fung Cheung, Chit Chow, Jimmy Yu Wai Chan

**Affiliations:** ^1^ Clinical Laboratory Gleneagles Hospital Hong Kong Hong Kong Hong Kong; ^2^ Department of Pathology University of Hong Kong Hong Kong Hong Kong; ^3^ Department of Anatomical and Cellular Pathology Chinese University of Hong Kong Hong Kong Hong Kong; ^4^ Chief of Division of Head and Neck Surgery and Division of Plastic and Reconstructive Surgery, School of Medicine University of Hong Kong Hong Kong Hong Kong

**Keywords:** CSF1, eosinophilia, Langerhans cells, Sclerosing mucoepidermoid carcinoma

## Abstract

**Key Clinical Message:**

When faced with a slowly enlarging firm mass in the parotid gland accompanied by a histological picture of unusual sclerosis with abundant Langerhans cells and eosinophilic infiltrates, sclerosing mucoepidermoid carcinoma with eosinophilia should be considered as one of the differential diagnoses. Further studies are warranted for accurate diagnosis and appropriate treatment.

**Abstract:**

Sclerosing mucoepidermoid carcinoma of the salivary gland with eosinophilia is a rare tumor mostly negative for the MAML2 rearrangement commonly seen in salivary mucoepidermoid carcinoma. It was not listed as an entity in the 2022 WHO Classification of Head and Neck Tumors. We presented one case initially diagnosed as Langerhans cell histiocytosis and recurred as a frankly invasive carcinoma. Molecular studies showed CSF1 gene derangement and provided new understanding concerning the Langerhans cell and eosinophilic reaction. Further molecular studies on this entity would throw light on its oncogenesis and refine its nomenclature.

## Graphical abstract

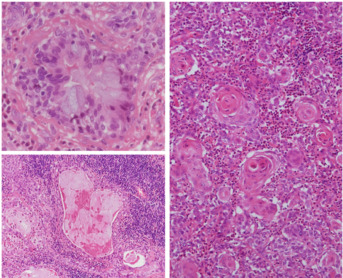

## 1 INTRODUCTION

Malignant salivary gland tumors characterized by mucoepidermal differentiation with sclerotic stroma rich in lymphocytes and eosinophils have been designated the name sclerosing mucoepidermoid carcinoma with eosinophilia^1^, ^2^, ^3^, ^4^ (SMECE). However, it has not been listed as an entity in the chapter on salivary gland, 2022 WHO Classification of Head and Neck Tumors.^5^ Some reports highlighted the lack of MAML2 translocation in these tumors, as distinct from classical mucoepidermoid carcinoma (MEC) of the salivary glands. Some argued against grouping them under MEC based on their variable morphological features and the lack of MAML2 translocation. This counterargument is supported by the prominence of keratinization in the squamoid component and relatively reduced glandular or intermediate cell component noted in SMECE, such that other entities, e.g., adenosquamous carcinoma should be considered in the differential diagnosis. The lack of a well‐documented molecular marker also makes categorizing SMECE as a distinct entity difficult. A same‐named tumor has been described in the thyroid.^6^ The thyroid SMECE lacks common thyroid cancer mutations nor MAML2 translocation according to studies by Shah et al.^7^ Whether SMECE of the head and neck region share similar histogenetic origin or molecular derangement requires further studies on larger tumor series. The underlying mechanism for the sclerotic stroma and eosinophilia has received little attention as these features could be seen in other tumors. We report a similar case in the parotid gland that was initially diagnosed as Langerhans cell histiocytosis due to the prominent Langerhans cell and eosinophilic reaction. It recurred 2 years later as a frank carcinoma fitting into the SMECE category by morphology. Molecular studies provided possible new understanding concerning the Langerhans cell and eosinophilic reaction.

## 2 CLINICAL HISTORY AND FINDINGS

The patient was a 66‐year‐old Chinese woman who presented in another hospital 3 years ago with a slowly enlarging left cheek swelling for a few months. The swelling measured about 3 cm in diameter, hard in consistency and was situated in the parotid gland on radiological examination. Partial parotidectomy was done and specimen (S1) was initially diagnosed as Langerhans cell histiocytosis associated with benign glandular proliferations focally extending to the margin. The regional lymph node sampled showed reactive changes with increased Langerhans cells. After 2 years, the patient noticed recurrent cheek swelling over the same site. Clinical‐radiological work‐up revealed a recurrent mass in the parotid gland associated with enlarged regional lymph nodes. Biopsy of the swelling showed frank carcinoma and was reported as sclerosing mucoepidermoid carcinoma with eosinophilia. The patient was then transferred to our hospital for further management. Radical parotidectomy sacrificing the lower branches of the facial nerve with reconstruction using the sural nerve, and regional lymph node dissection were performed. The specimen was subsequently sent for pathological study. Post‐operative external radiotherapy to the neck was administered and the patient was free from disease 6 months after operation.

## 3 MATERIALS AND METHODS

Formalin‐fixed‐paraffin‐embedded H & E sections were made from the radical parotidectomy specimen in the usual manner. Immunostainings for AE1/3, p16, p40, S100, and Ki67 were performed using the Dako Omnis and Leica Bond‐III automated IHC systems, and immunostaining for CD1a used the Leica Bond‐III automated IHC system.

Representative sections were subjected to CSF1 break‐apart FISH test, with FISH probes targeting the 5'‐end and 3'‐end of the CSF1 locus labeled orange and green respectively (Empire Genomics). Any fusion transcripts of the specimen were studied by the Illumina RNA pan‐cancer RNA‐seq panel (Illumina), which covered the fusion transcripts of 1385 cancer‐related genes including the CSF1 gene. The expression of the CSF1 gene was detected by RNAscope™ Probe‐ Hs‐CSF1 (Advanced Cell Diagnostics).

## 4 RESULTS

**FIGURE 1:**
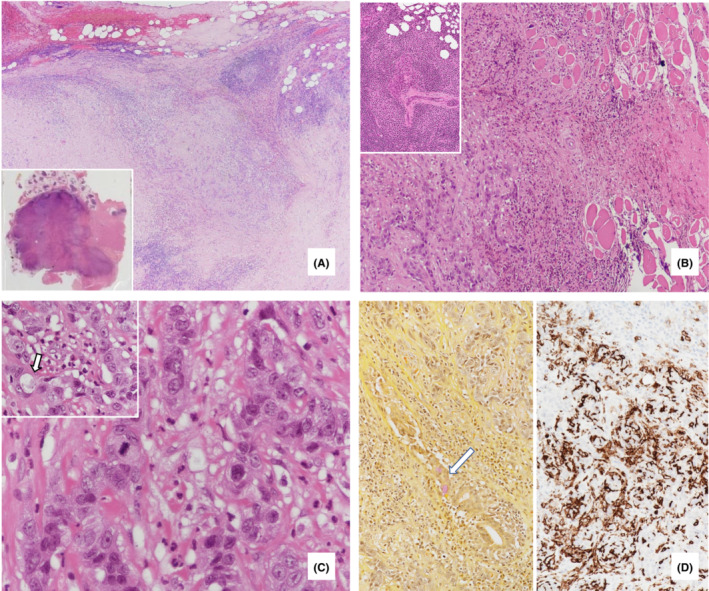
Recurrent tumor (S2) (A). Tumor with prominent fibroinflammatory background. Inset: Tumor infiltrating adjacent parotid gland and muscle; (B). Cords and trabeculae of carcinoma cells infiltrating skeletal muscle. Inset: Benign lymphoepithelial lesion in adjacent salivary gland; (C). Poorly differentiated carcinoma cells with pleomorphic nuclei, prominent nucleoli and occasional mitosis. Inset: Abortive lumen (white arrowed) in some cell clusters; (D). Left: Mucicarmine stain highlighted mucinous cells (white arrowed). Right: CD1a immunostain highlighted abundant Langerhans cells in the background.

Gross and microscopic examination of the radical parotidectomy specimen (S2) showed a 4 cm high‐grade carcinoma (AFIP grading for MEC) invading salivary gland, skeletal muscle, and trapping hypertrophic nerves (Figure 1A,B). The tumor consisted of solid lobules, cords, and trabeculae of moderate to highly pleomorphic carcinoma cells with focal keratinization (Figure 1C). Primitive glandular differentiation with foamy cells and abortive lumens staining positive for mucicarmine (Figure 1D) could be focally seen. Atypical mitoses (5/10 HPF) and necrosis were present. Immunohistochemical studies showed tumor cells positive for cytokeratin AE1/3, p63, and p40; negative for S100. Ki67 proliferative index varied from 15% to 30%. There was marked stromal sclerosis forming fibrous strands and bands. Heavy chronic inflammatory infiltrates including many Langerhans cells (CD1a positive, Figure 1D) and eosinophils were present in the background. The adjacent salivary gland showed non‐sclerosing chronic sialadenitis with lobular lymphocytic infiltrates and scanty lymphoepithelial lesions (Figure 1B). This high‐grade carcinoma with mucoepidermoid differentiation and the accompanying features fit into the published criteria of SMECE. Ten regional lymph nodes dissected showed no malignancy.

**FIGURE 2:**
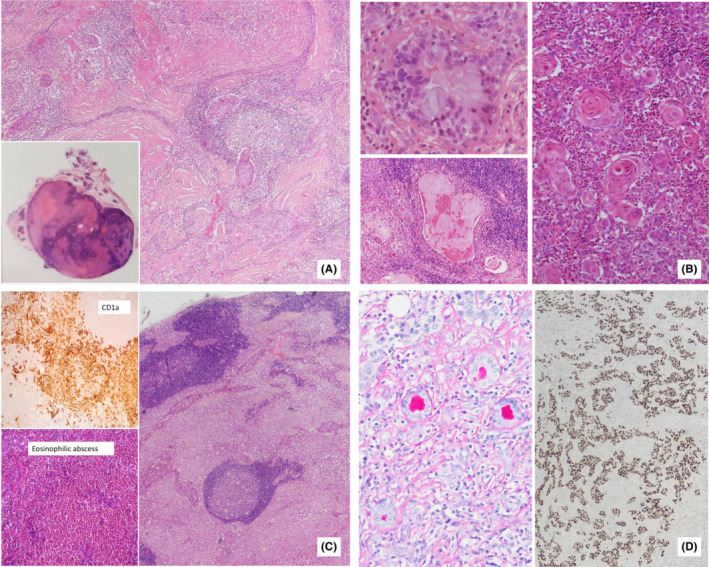
Primary tumor (S1) (A). Small lobules and branching cords of a low grade carcinoma in sclerotic stroma. Inset: Well‐circumscribed tumor in parotid gland with rich lymphocytic infiltrates; (B). Right: Keratinised tumor lobules forming keratin pearls among heavy eosinophilic infiltrates, Left upper: Ductal differentiation lined by mildly pleomorphic columnar mucinous cells, Left lower: Focal cystic structures containing secretion; (C). Mimic of Langerhans cell histiocytosis by sheets of pinkish Langerhans cells (right), CD1a immunostain positive (left upper) and eosinophilia forming abscesses (left lower); (D). Well‐differentiated ductal structures with PAS diastase resistant luminal content (left), p63 immunostain highlighted the infiltrative strands of carcinoma cells (right).

Slide review of the initial excisional specimen (S1) was performed and showed a well‐circumscribed low‐grade (AFIP grading) carcinoma hiding within a fibroinflammatory background (Figure 2A) and focally touching resection margin. Compared with S2, there was a similar SMECE picture but much less aggressive. It consisted of lobules and cords of squamoid cells with keratinization and minimal nuclear pleomorphism (Figure 2B). Cystic change and ductal differentiation with well‐formed lumen positive for PAS (diastase resistant, Figure 2B,D) lined by foamy columnar mucinouscells were focally seen. Mitosis numbered about 1–2/10 HPF. There was no evidence of IgG4‐associated disease. The immunohistochemistry profile (Figure 2D) was the same as that in S2 (Ki67 not available). There were distinctive sheets of Langerhans cells and eosinophilia forming eosinophilic abscesses mimicking Langerhans cell histiocytosis (Figure 2C). Definite tumor progression on recurrence from low (S1) to high‐grade carcinoma (S2) could be demonstrated with respect to cellular pleomorphism, necrosis, tumor differentiation, and invasiveness. The AJCC pathological staging also progressed from pT2 to pT4a.

**FIGURE 3:**
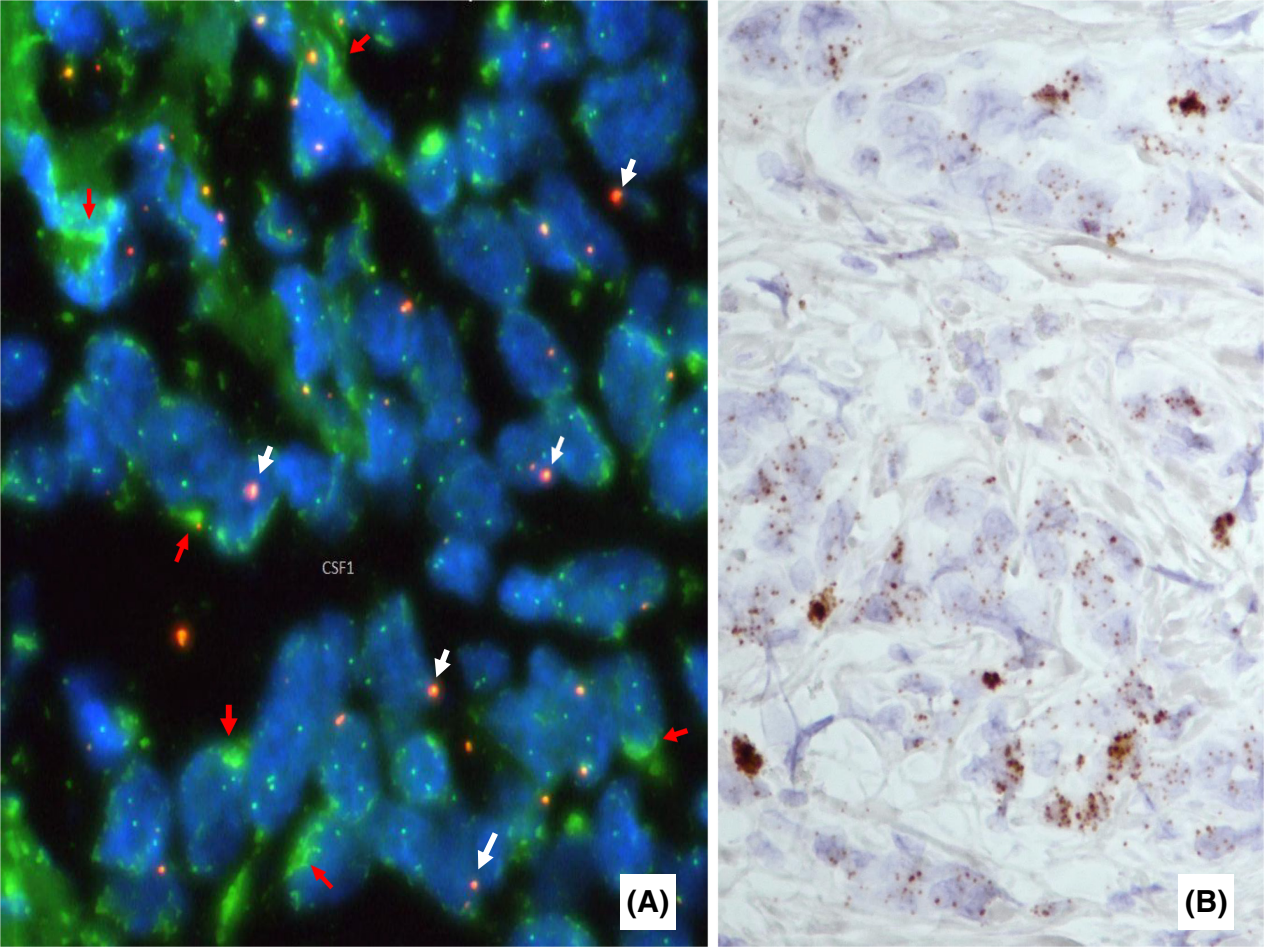
Molecular studies: (A). FISH showed single orange‐red dot of normal CSF1 gene (white arrowed) and lumpy green dots of duplicated CSF1 3'end (red‐arrowed) in most tumor cells; (B). RNAscope showed expression of CSF1 (brown granules) in pleomorphic carcinoma cells.

FISH using break‐apart probe for CSF1 was performed to detect possible derangement of the CSF1 gene with subsequent CSF1 over‐expression. In most tumor cells there was 1 copy of the normal gene (fused green and orange signal giving orange‐red color), and multiple green signals signifying duplicated CSF1 3' end with deletion of the 5' end in the other chromosome (Figure 3A). Illumina TruSight RNA pan‐cancer assay detected no fusion transcripts in the specimen. On the other hand, the expression of the CSF1 gene in tumor cells could be demonstrated by RNA in‐situ hybridization (RNA scope) as coarse and fine granules (Figure 3B).

## 5 DISCUSSION

The present case highlighted the difficult diagnoses in anatomical pathology of a sclerosing salivary gland lesion accompanied by lymphohistiocytic infiltrates and eosinophilia. Differential diagnoses included Langerhans cell histiocytosis, fibroinflammatory conditions such as IgG4‐associated disease, benign lymphoepithelial lesion, and necrotizing sialometaplasia. Malignant tumors obscured by the fibroinflammatory background should also be considered. An accurate and early diagnosis with appropriate treatment would certainly improve prognosis and mitigate patient morbidity.

SMECE of the salivary gland is extremely rare, numbering about 12 in literature reviews.^2^, ^4^, ^8^ It is not a well‐defined entity and was not listed in the 2022 WHO Classification of Head and Neck Tumors. According to most reports, tumors falling into this category showed mucoepidermoid differentiation with low‐grade cytology and a sclerotic background with heavy eosinophilic infiltrates, without mention of Langerhans cells. No molecular markers have been found associated with this tumor, except for the negative MAML2 rearrangement. A host of molecular defects have been demonstrated in ‘MAML2 translocation‐negative mucoepidermoid carcinoma’ in one study,^9^ but there was no mention of this SMECE subtype. Thus, the true nature and nomenclature of this group of tumors await further clarification.

The present case with the abundant Langerhans cells masquerading as Langerhans cell histiocytosis triggered the search for an underlying mechanism. Our studies were inspired by the findings of CSF1 rearrangement and its overexpression in tenosynovial giant cell tumors (TSGCT), attracting dendritic cells including Langerhans cells (CSF1 receptor+) to migrate into the mesenchymal tumor and proliferate.^10^, ^11^ Instead of CSF1 gene rearrangement, we detected increased CSF1 (3'end) copy number with deletion of the 5′ end, coupled with increased expression of CSF1 in tumor cells on RNA scope. Over‐expression of CSF1 without documented translocation has also been found in a proportion of TSGCT.^12^ CSF1 over‐expression is the most likely cause of the marked Langerhans cell reaction, leading to subsequent eosinophilia and sclerosis. Whether this molecular defect with subsequent ‘landscape effect’ was an isolated event, or a significant tumor marker merits further studies on this group of tumors.

## 6 CONCLUSION

SMECE of the salivary gland is a rare entity that could be easily missed on biopsy due to the masquerading effect of the company it keeps. Sclerosing background, abundance of eosinophils, and Langerhans cells would prompt the anatomical pathologist to look for an underlying tumor. Further studies on a larger series are needed to define its histogenesis, possible molecular defect, and succinct categorization for better patient management and research.

## AUTHOR CONTRIBUTIONS

**Florence Man Fung Cheung:** Conceptualization; data curation; formal analysis; investigation; methodology; validation; writing – original draft; writing – review and editing. **Chit Chow:** Data curation; formal analysis; investigation; methodology; validation. **Jimmy Yu Wai Chan:** Data curation; investigation; methodology; validation.

## Data Availability

All data are available for review if required.
